# Large Non-enhancing Breast Cancer on Breast Magnetic Resonance Imaging: A Case Report

**DOI:** 10.7759/cureus.2332

**Published:** 2018-03-15

**Authors:** Johannes Peters, Wei Che Tsai, Gudrun Peters

**Affiliations:** 1 Medical School, University of Tasmania; 2 Radiology, Royal Hobart Hospital; 3 Radiology, Regional Imaging, Tasmania, I-Med Network, Australia

**Keywords:** breast cancer, non-enhancing, breast mri

## Abstract

A 55-year-old female presented with vague symptoms in the lateral left breast. Digital breast tomosynthesis and breast ultrasound showed no focal lesion, and magnetic resonance imaging (MRI) was subsequently performed. No suspicious enhancement was seen on MRI; in particular, no suspicious lesion was seen in the area of clinical concern. In view of persisting focal mastalgia and vague parenchymal changes in the symptomatic area on repeat targeted ultrasound, a core biopsy was performed. Final pathology after left mastectomy with axillary clearance showed a 42 mm grade 2 invasive ductal carcinoma. Ten out of 15 lymph nodes contained metastatic carcinoma. This case report presents a large ductal breast cancer with no enhancement on breast MRI. Factors that may contribute to the non-detection of breast cancers on MRI studies will be discussed.

## Introduction

Since contrast-enhanced breast magnetic resonance imaging (MRI) was introduced in 1986 [1], its application in the detection and evaluation of breast cancer has been intensely researched. As an adjunct to clinical examination, mammography, and ultrasound, MRI has been shown to be highly sensitive in identifying breast malignancies with reported ranges from 90%-96% [2-3]. The principle of tumor angiogenesis had previously led radiologists to assume that non-enhancing breast lesions were not malignant [4-6]. However recent studies have shown that MRI cannot be used as a single imaging modality for the evaluation of breast cancer, as false negative results can occur [2-5,7]. 

We present a case of a large breast cancer that was non-enhancing on breast MRI.

## Case presentation

A 55-year-female patient presented to a private radiology practice with vague discomfort in the lateral left breast. Digital breast tomosynthesis (DBT) with 2D reconstructed mammographic views was obtained using a Hologic Selenia Dimensions AWS 8000 System (Hologic, Bedford, Conn, USA). Minor architectural distortion was seen in the left craniocaudal (CC) view only and the mammogram was sent for a second opinion.

A bilateral breast ultrasound was performed on the same day as the DBT, for further assessment per standard practice, using a Philips Epiq 7 machine with an 18-5 MHz transducer (Philips, Yorba Linda, CA, USA). Ultrasound findings revealed widespread bilateral shadowing, which was presumed normal for this patient, due to Cooper’s ligaments and dense fibrous stromal tissue. No suspicious lesion was present.

**Figure 1:**
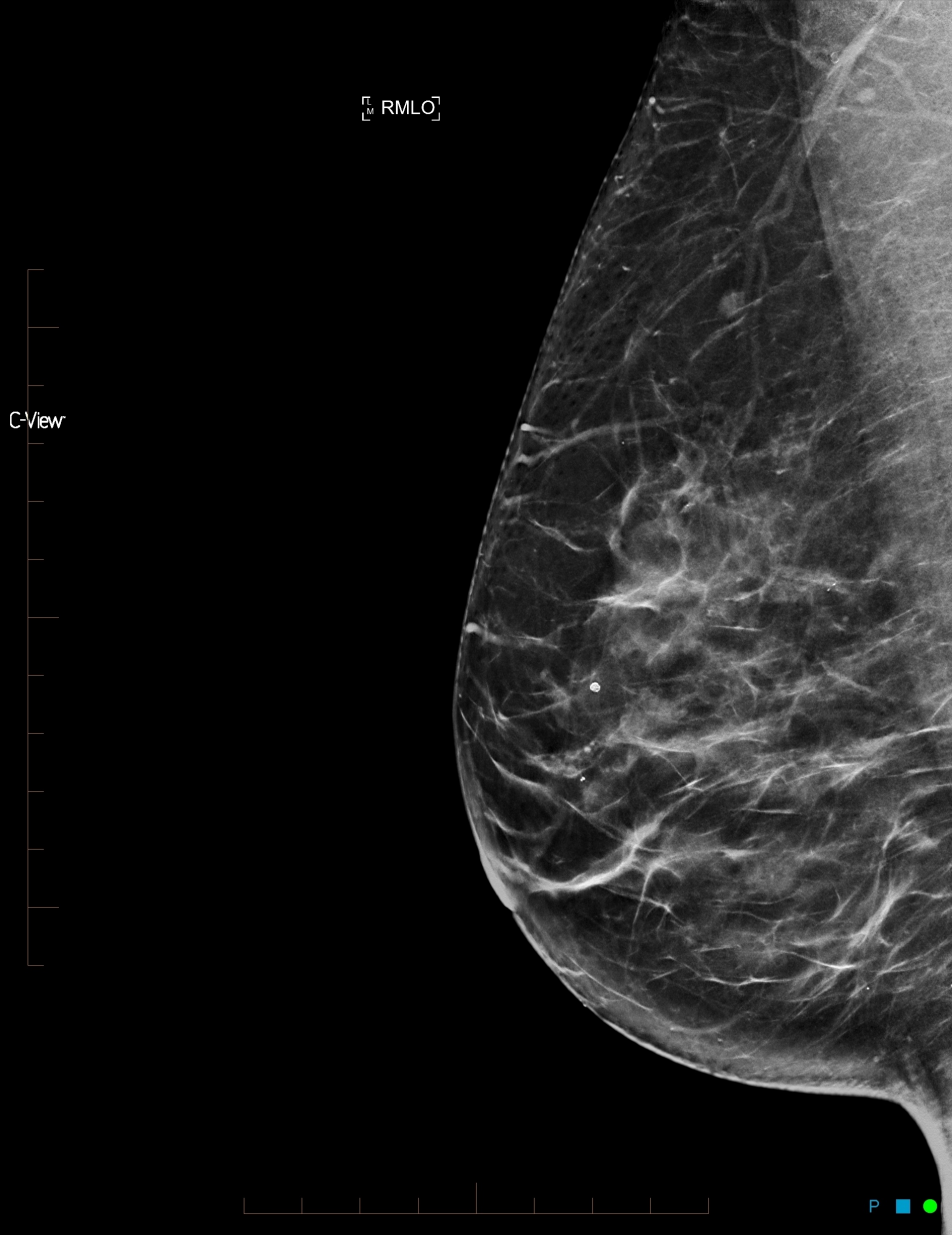
Synthesized mammogram right mediolateral oblique view

**Figure 2:**
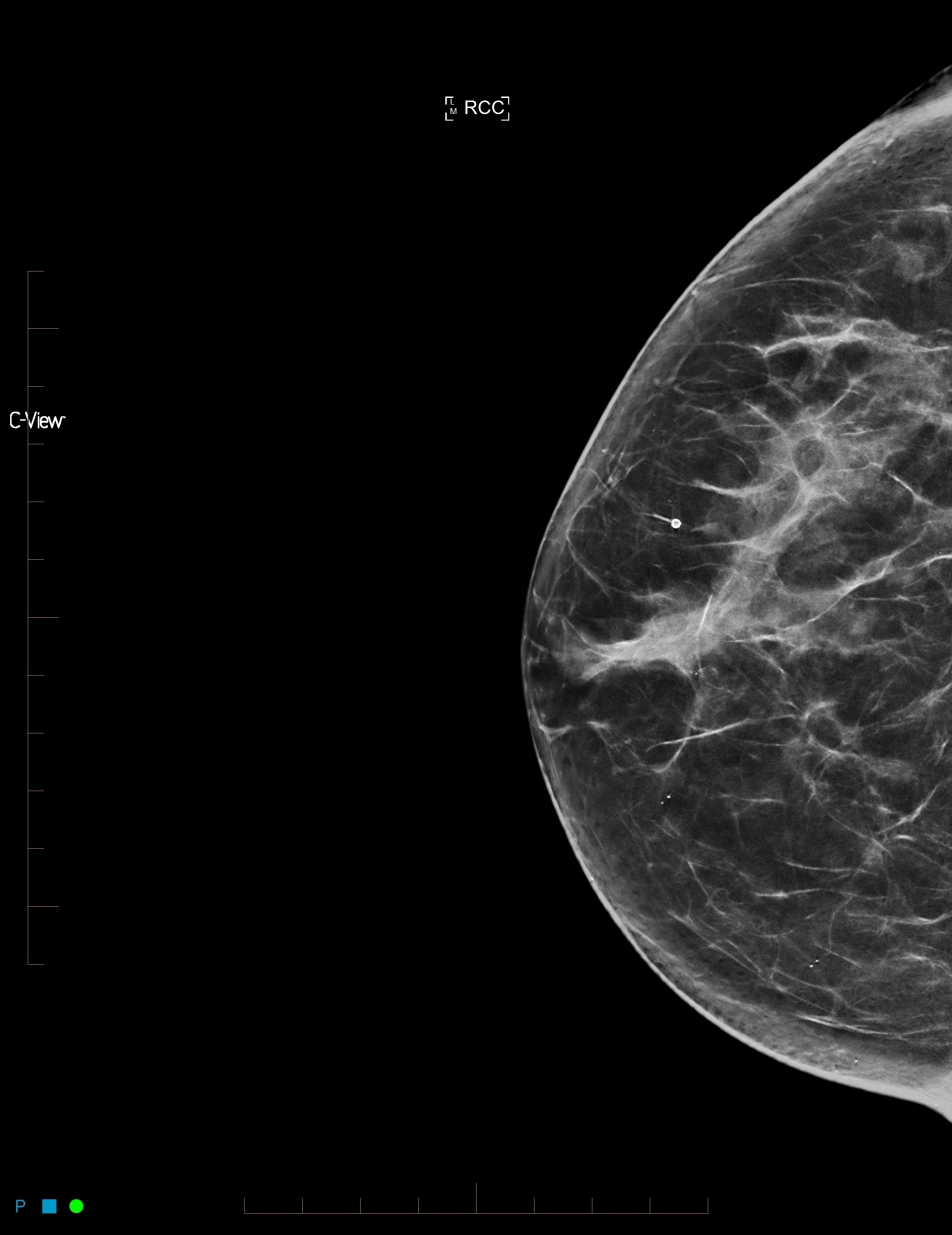
Synthesized mammogram right craniocaudal view

**Figure 3:**
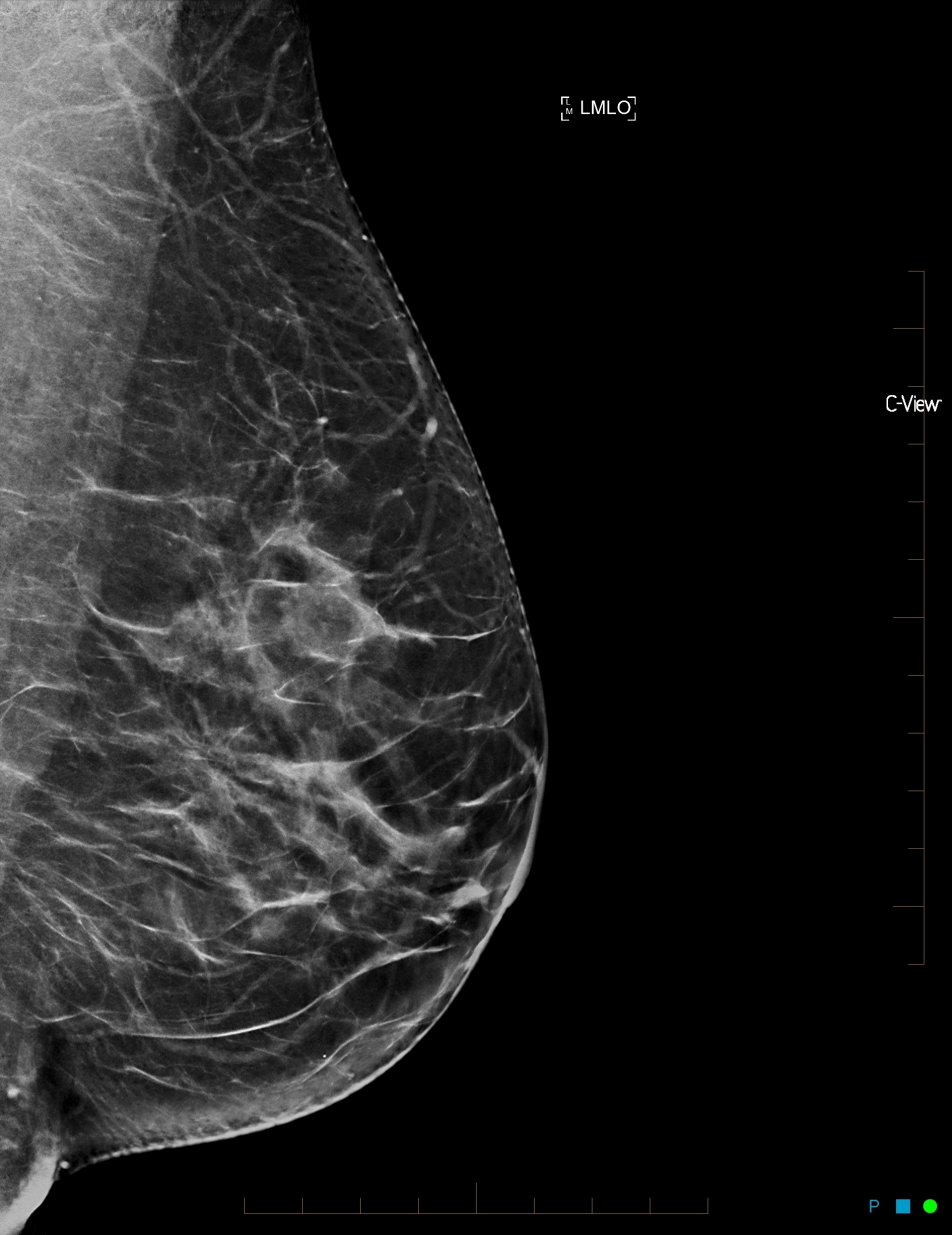
Synthesized mammogram left mediolateral oblique view

**Figure 4:**
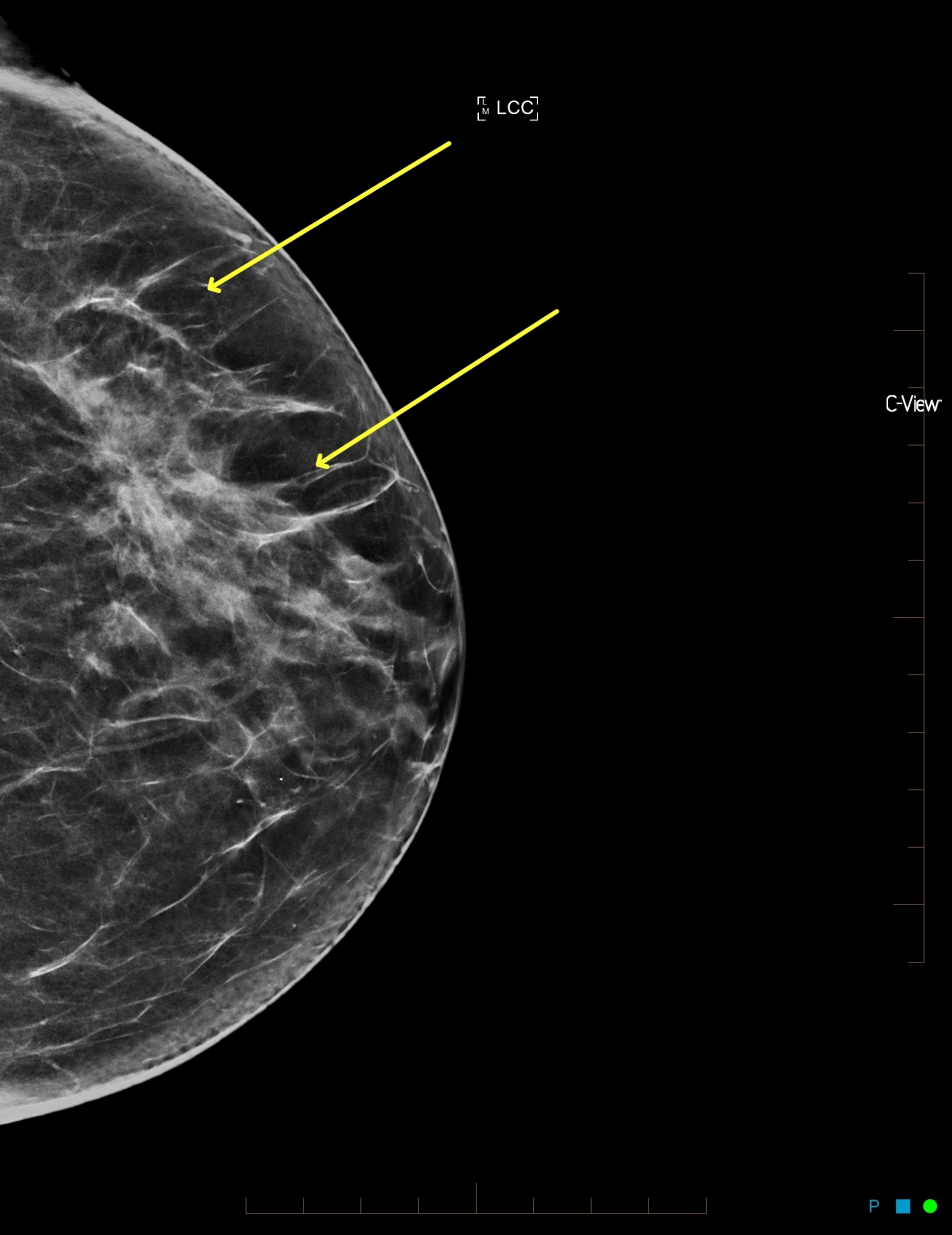
Synthesized mammogram left craniocaudal view with minor architectural distortion in the left outer breast

After reviewing the initial mammogram (Figures 1-4), it was decided to recall the patient for a possible subtle left lateral architectural distortion, that was only seen in the craniocaudal view (Figure 4).

**Figure 5:**
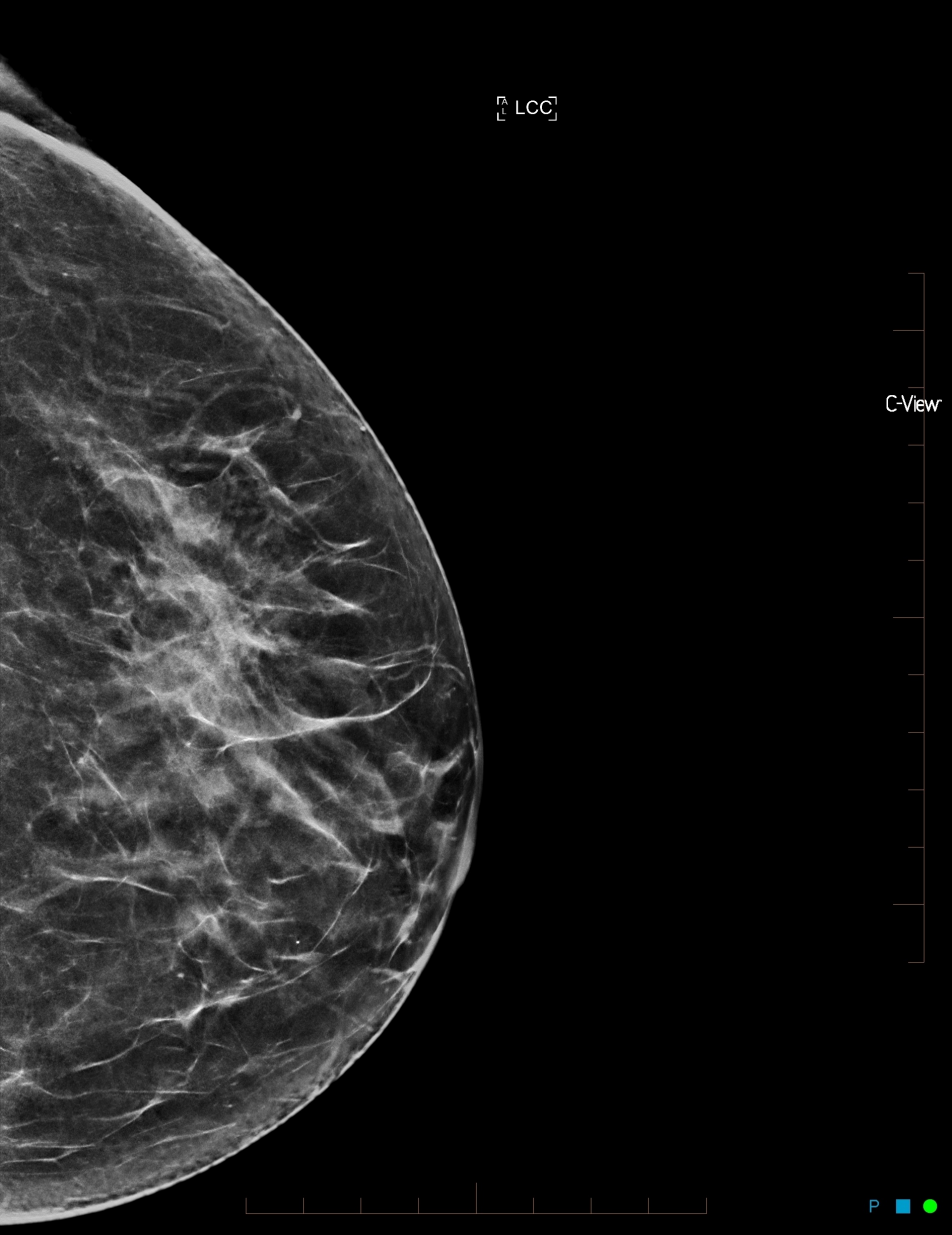
Synthesized mammogram left craniocaudal repeat view with resolution of the minor architectural distortion in the left outer breast

A repeat CC view was performed and the architectural distortion resolved (Figure 5).

**Figure 6:**
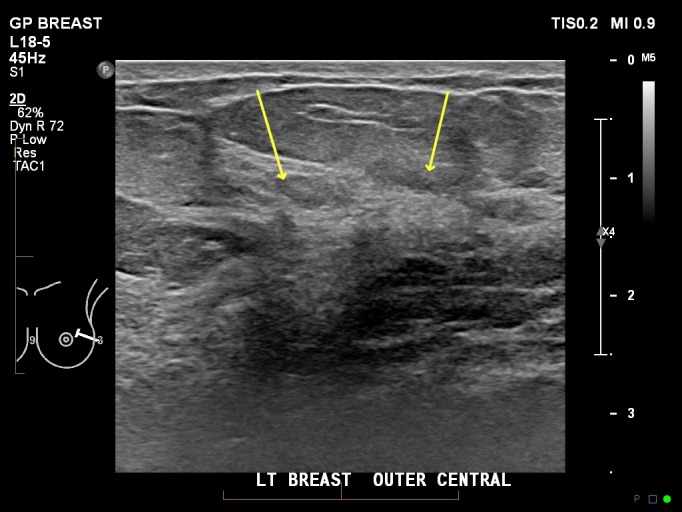
Ultrasound left outer central breast showing dense glandular tissue with minimal shadowing

The targeted ultrasound showed slightly increased shadowing without definable margins in the lateral left breast (Figure 6), which was asymmetric when compared with the contralateral side. The cortex of one left axillary lymph node (LN) showed minimal eccentric thickening, but the cortical thickness was still within normal limits according to ultrasound criteria (less than 3 mm) [8].

Due to the indeterminate nature of the left breast lesion, MRI was proposed as a problem-solving tool.

**Figure 7:**
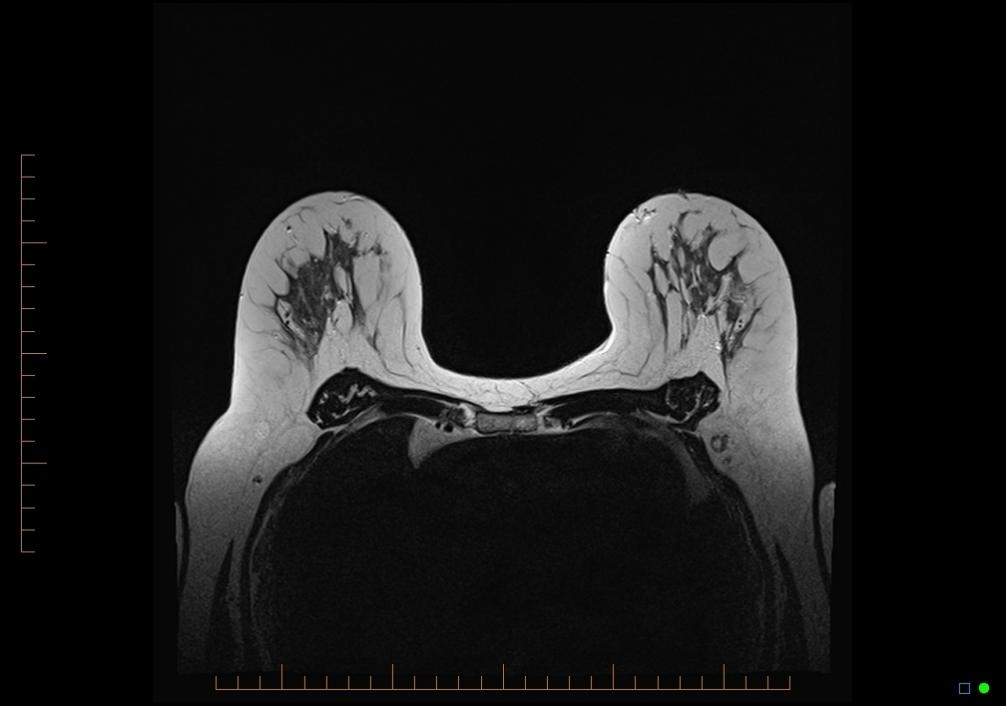
MRI breast: T2 axial, no suspicious axillary lymph nodes MRI: magnetic resonance imaging

A breast MRI study was undertaken on a 1.5T Magnetom Espree scanner (Siemens, Erlangen, Germany) with a dedicated eight-channel breast coil. T1-weighted coronal pre-contrast images were obtained to assess the axillary and supraclavicular LN (slice thickness: 4 mm). Pre-contrast T2-weighted turbo spin echo sequences (Figure 7) were carried out (slice thickness: 3 mm). A dynamic T1-weighted study with fat suppression before and four times after administration of 0.1 mmol kg-1 of gadolinium-DTPA was performed (slice thickness: 1.2 mm, acquisition time per sequence 90 s). Post-processing protocols included obtaining subtraction and maximum intensity projection (MIP) images.

**Figure 8:**
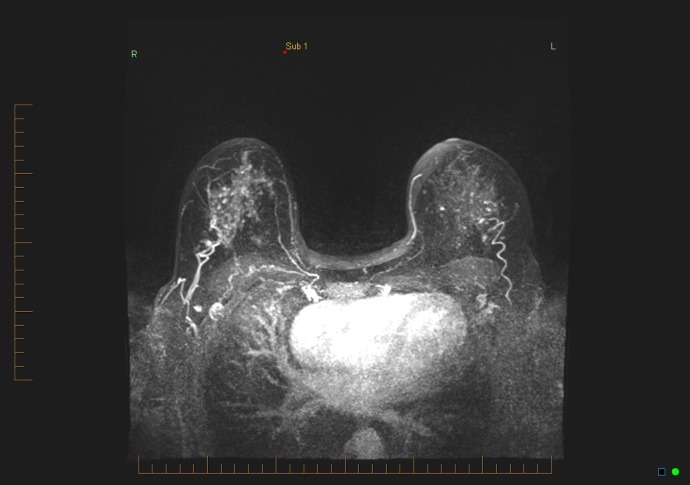
Maximum intensity projection (MIP) subtraction 1

**Figure 9:**
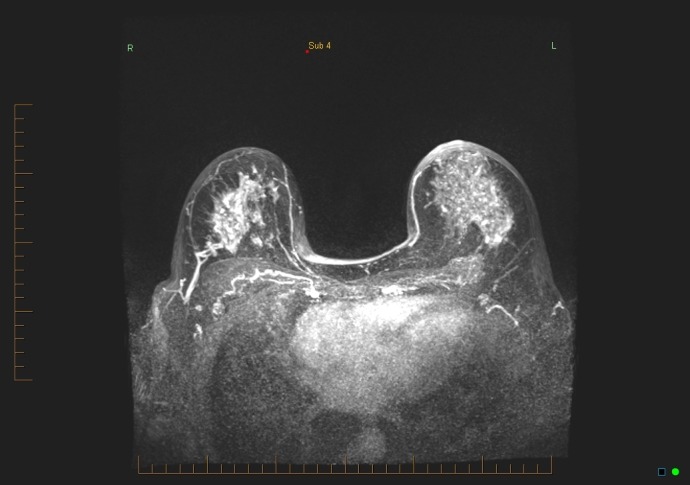
Maximum intensity projection (MIP) subtraction 4

There was mild to moderate background enhancement of the breast parenchyma on the MRI study, with no suspicious enhancement seen in either breast. Specifically, no mass or non-mass enhancement was seen in the lateral left breast (Figures 8-9). There was no suspicious LN enhancement.

Persistence of focal breast discomfort in the lateral left breast, combined with the vague shadowing seen in the area on ultrasound, led to the decision to perform a core needle biopsy in the area. A fine needle aspirate (FNA) of the slightly prominent left axillary LN was carried out at the same time.

Histopathology of the core biopsy showed a provisional grade 2 infiltrating ductal carcinoma, not otherwise specified (NOS) associated with scattered foci of solid intermediate grade ductal carcinoma in-situ (DCIS). A cytology analysis of the left axillary LN FNA demonstrated malignant features consistent with metastatic adenocarcinoma.

The patient proceeded to surgical treatment with mastectomy and axillary clearance. The final pathology result showed a 42 mm grade 2 invasive ductal carcinoma with 10 out of 15 lymph nodes containing metastatic carcinoma.

## Discussion

MRI has been shown to have high sensitivity for breast cancer detection [2,4,7], but false negatives do still occur. Technical difficulties, reader perception, and lesion characteristics are components that may be implicated in the non-detection of breast cancers on MRI studies.

There are several technical difficulties that may arise when imaging the breast with MRI. Delayed contrast uptake of the lesion may occur due to the suboptimal timing of the post-contrast sequences, small cannula size, and/or the reduced cardiac output of the individual patient. The positioning of the patient is crucial in MRI studies of the breast, as inadequate positioning can result in the lesion being outside the coil and therefore not captured in the study [6,9]. A motion artefact may obscure small lesions, and therefore it is important to gently fixate the breasts and advise the patient to remain still during the scan [3-4,7]. Strong background enhancement can be problematic, as normal glandular tissue may show the same enhancement pattern as breast cancer [2,4-5].

No technical difficulties occurred in this case, the timing of the study and patient positioning were optimal, background enhancement was rated as mild to moderate but did not affect the area of interest, and the study was not impaired by a motion artefact.

The second category of potential causes for false negatives involves the readers of the MRI studies. During image interpretation, errors can occur, including lesions being missed or incorrectly identified [4]. There may also be incorrect recommendations for management [2-3,7,9]. Our practice’s standard protocol includes a double reading of breast MRI studies by breast radiologists. The MRI result, in this case, was considered concordant by two breast radiologists: “No suspicious mass or non-mass enhancement was seen on the study, especially in the left outer central breast, no suspicious lymph nodes were noted” (Figures 8-9).

Several breast cancer characteristics that are associated with non-enhancement on breast MRI have been described in the literature. These include small size, the location of the lesion, and histological subtype [2,9]. Small lesions less than 3 mm may be supplied with nutrients exclusively through diffusion. In these lesions, no tumor angiogenesis is required and therefore no enhancement occurs on MRI [4-5]. This explains false-negative results in cases of small, invasive carcinomas [3,5].

Tumor enhancement adjacent to normally enhanced structures, such as vessels or the nipple, may lead to missed small lesions [4-5]. Additionally, tumors directly adjacent to the chest wall may show less signal intensity and be classified as benign [5].

Various histological subtypes of breast cancer have been described as sometimes presenting with non-enhancement or late enhancement on breast MRI. These include lobular, mucinous, and tubular cancers [10]. Furthermore, the degree of angiogenesis is variable such as in the case of DCIS, which expresses less vascular endothelial growth factors and therefore has decreased angiogenesis [4].

## Conclusions

In the case presented, a large 42 mm grade 2 invasive ductal carcinoma NOS did not show enhancement in the MRI study. Technical difficulties, reader interpretation error, and causes due to tumor type could all be excluded. No explanation for why this large cancer did not show MRI enhancement can be given.

In conclusion, false negatives occur on breast MRI. The authors hope to demonstrate that even if there is no significant imaging evidence of a malignant lesion, further investigations should still be performed if there is clinical doubt.
